# Preparation of superhydrophobic surfaces with micro/nano alumina molds

**DOI:** 10.1039/c8ra07497f

**Published:** 2018-10-30

**Authors:** Takashi Yanagishita, Kaito Murakoshi, Toshiaki Kondo, Hideki Masuda

**Affiliations:** Department of Applied Chemistry, Tokyo Metropolitan University 1-1 Minamiosawa Hachioji Tokyo 192-0397 Japan yanagish@tmu.ac.jp

## Abstract

Polymer micro/nano hierarchical structures were successfully formed by photo-nanoimprinting using anodic porous alumina molds. Anodic porous alumina molds with hierarchical structures were prepared by the anodization of an Al substrate with a micro-concave array. The obtained surfaces with hierarchical structures exhibited superhydrophobicity. The hydrophobic properties of the obtained samples were dependent on the surface structures and could be optimized by changing the micro- and nanopatterns in the hierarchical structure.

## Abstract 2

Superhydrophobic surface with hierarchical structures prepared by nanoimprinting using anodic porous alumina molds.

## Graphical abstract

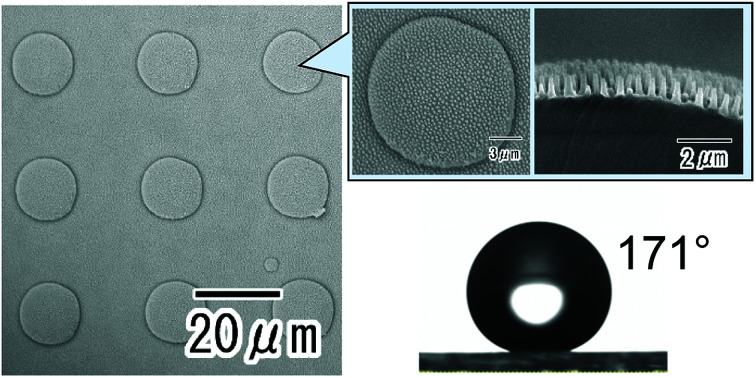

## Introduction

There has been increasing interest in the preparation of various functional surfaces based on bio-inspired approaches. Among them, studies on the preparation of superhydrophobic surfaces inspired by naturally occurring micro- or nanostructures have been intensively carried out.^1,2^ A large number of works have been reported on the preparation of superhydrophobic surfaces by imitating the structures on the surfaces of plants or animals, such as lotus leaves and the legs of water striders.^3–13^ The realization of superhydrophobicity by these approaches is based on the preparation of fine structures on the scale of micro- and nanometers. Hierarchical structures composed of units with different dimensions are well known to be particularly effective for achieving superhydrophobicity. A large number of works have been reported on the preparation of hierarchical structures providing hydrophobicity.^14–16^ The preparation process must satisfy the following requirements. First, it must allow precise control of the fine structures on the surface because the hydrophobicity is strongly dependent on the geometrical structure. In addition, the process must possess the capability for high-throughput processing. A nanoimprinting process, which generates fine patterns of a polymer using a mold, is a promising candidate for the preparation of fine patterns because it satisfies the above-mentioned requirements.^17,18^ Previously, we reported a nanoimprinting process using anodic porous alumina molds.^19–21^ Anodic porous alumina, which is formed by the anodization of Al in an acidic electrolyte, is a typical self-ordered material.^22^ The use of anodic porous alumina as a mold for nanoimprinting allows the formation of fine patterns over a large sample area owing to its naturally occurring self-ordered nanostructures. The hierarchical structures can also be prepared by using a mold composed of micro/nano structures, which is formed by anodization of Al with patterns of micro scales.^23^ In the present report, we describe the preparation of the micro/nano hierarchical structures for super-hydrophobic surfaces by a nanoimprinting process using an anodic porous alumina mold. In the process, an anodic porous alumina mold with micro/nano hierarchical structures was prepared through the anodization of Al with patterned structures on the micron scale. Using the obtained porous alumina as a mold for nanoimprinting, hierarchical polymer structures with superhydrophobicity were prepared effectively. Although there have been some reports on process for preparing superhydrophobic surfaces using anodic porous alumina as a starting structure, a process that can generate the superhydrophobic surface with precisely controlled surface geometrical structures has not been established so far.^24,25^ The present process can be formed the precisely controlled hierarchical structures on a surface of substrates by nanoimprinting using anodic porous alumina molds. By optimizing the hierarchical structure of anodic porous alumina molds, the preparation of superhydrophobic surface having a contact angle greater than 170° could be realized. In addition, the present process is applicable to the high-throughput preparation of large superhydrophobic surfaces. The obtained superhydrophobic surfaces are expected to be used in various application fields requiring the outstanding hydrophobicity over a large area.

## Experimental

**Fig. 1:**
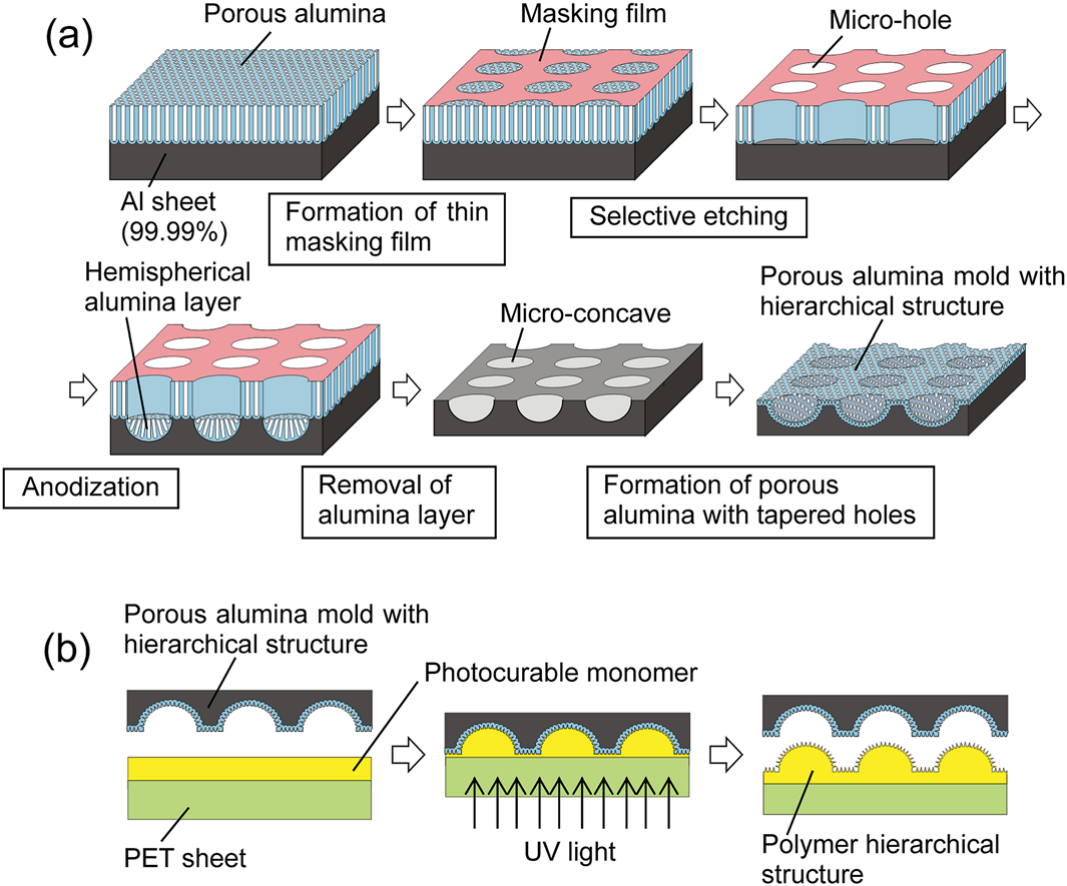
(a) Schematic illustration of the preparation of anodic porous alumina mold with hierarchical structures. (b) Schematic illustration of the preparation of polymer nano/micro hierarchical structures on the surface of a substrate by nanoimprinting using an anodic porous alumina mold.

Fig. 1 shows a schematic illustration of the preparation of micro/nano hierarchical structures by nanoimprinting using an anodic porous alumina mold. This process is composed of two steps: the preparation of a porous alumina mold with hierarchical structures, shown in Fig. 1(a), and the formation of a superhydrophobic surface by nanoimprinting using the anodic porous alumina mold, shown in Fig. 1(b). An Al sheet (99.99% purity) was used as the starting material for the porous alumina mold. Prior to the anodization, the Al sheet was electropolished using a mixed solution of perchloric acid and ethanol. The electropolished Al sheet was then anodized in 0.3 M oxalic acid solution at 17 °C under a constant voltage of 40 V for 20 min. To form the micropattern on the surface of the Al sheet, a thin resist mask with a microhole array structure was formed by a contact printing technique using a polydimethylsiloxane (PDMS) stamp.^23^ In this process, a thin polychloroprene rubber film was formed on the surface of the PDMS stamp by dip-coating in a 0.75 wt% toluene solution of polychloroprene and then transferred onto the surface of the anodic porous alumina through physical contact and detachment. The alumina microhole array was formed by selective etching of the anodic porous alumina with the resist mask in a mixed solution of chromic acid and phosphoric acid at 50 °C for 15 min. After the etching treatment, the sample was anodized again in 0.3 M oxalic acid solution at 40 V for 3 h. The second anodization generated a hemispherical oxide layer at the bottom of the alumina microholes. Micro-concave array structures were formed on the surface of the Al substrate by removal of the oxide layer using a mixed solution of chromic acid and phosphoric acid. The textured Al sheet was anodized again to form a nanohole array structure on the surface of the micro-concave array. In this study, the shape of the holes in the anodic porous alumina was controlled to a tapered shape using a combination of anodization and pore-widening treatment.^26,27^ The first anodization was performed in 0.1 M phosphoric acid under a constant voltage of 195 V at 0 °C for 3 min. The sample was then dipped in 10 wt% phosphoric acid at 30 °C for 25 min. Four repetitions of the anodization and wet etching generated tapered holes. The obtained anodic porous alumina molds were pretreated with a fluoroalkylsilane solution (Optool DSX, Daikin Industries Ltd.) to form the releasing layer for nanoimprinting.

Photo-nanoimprinting using a photocurable monomer was adopted to prepare polymer hierarchical structures on the surface of a substrate. In this experiment, a commercially available photocurable monomer (PAK-02A, Toyo Gosei) was used for the nanoimprinting. Although the details of composition of this monomer are not disclosed, it is a kind of monomer for acrylic resin. Micro/nano hierarchical structures were prepared on a polyethylene terephthalate (PET) sheet by nanoimprinting with the anodic porous alumina mold. A Ni layer of 5 nm thickness was deposited on the surface of the polymer fine pattern using an Ar ion beam sputtering apparatus to facilitate the formation of hydrophobic species on the surface. Hydrophobic treatment of the samples was carried out using a fluoroalkylsilane solution (Optool DSX, Daikin Industries Ltd.). The obtained samples were observed by scanning electron microscopy (SEM; JEOL JSM-6700F). The hydrophobicity of the samples was measured with a contact angle meter (Kyowa Interface Science; DM-300).

## Results and discussion

**Fig. 2:**
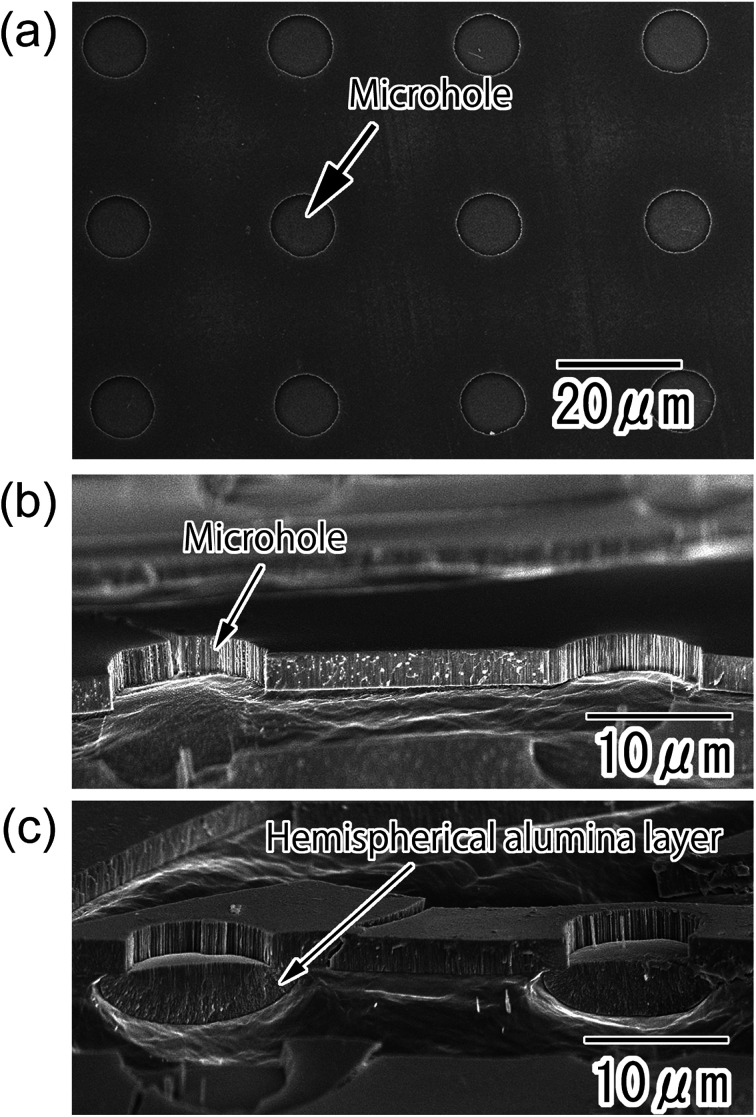
(a) Surface SEM image of alumina microhole array formed by the selective etching of anodic porous alumina with the resist mask. (b) Cross-sectional SEM image of the alumina microhole array. (c) Cross-sectional SEM image of the hemispherical alumina layer formed at the bottom of the microholes by the second anodization.

Fig. 2(a) and (b) show SEM images of the anodic porous alumina with a polychloroprene mask on its surface after the etching treatment. Microholes of 10 μm diameter were arranged with a 30 μm period over the sample (Fig. 2(a)). The diameter and period of the microholes were in good agreement with the surface pattern of the PDMS stamp. The cross-sectional image shown in Fig. 2(b) revealed that the alumina layer was dissolved selectively in the areas corresponding to the openings of the resist mask by the etching treatment. Subsequently, the bare Al was exposed at the bottom part of the microholes. Fig. 2(c) shows a cross-sectional SEM image of the sample after the second anodization. A hemispherical oxide layer was formed at the bottom of the microholes by the second anodization owing to the radial growth of anodic porous alumina at the bottom of the microholes. The size and shape of the hemispherical alumina layer could be controlled by adjusting the anodization time.

**Fig. 3:**
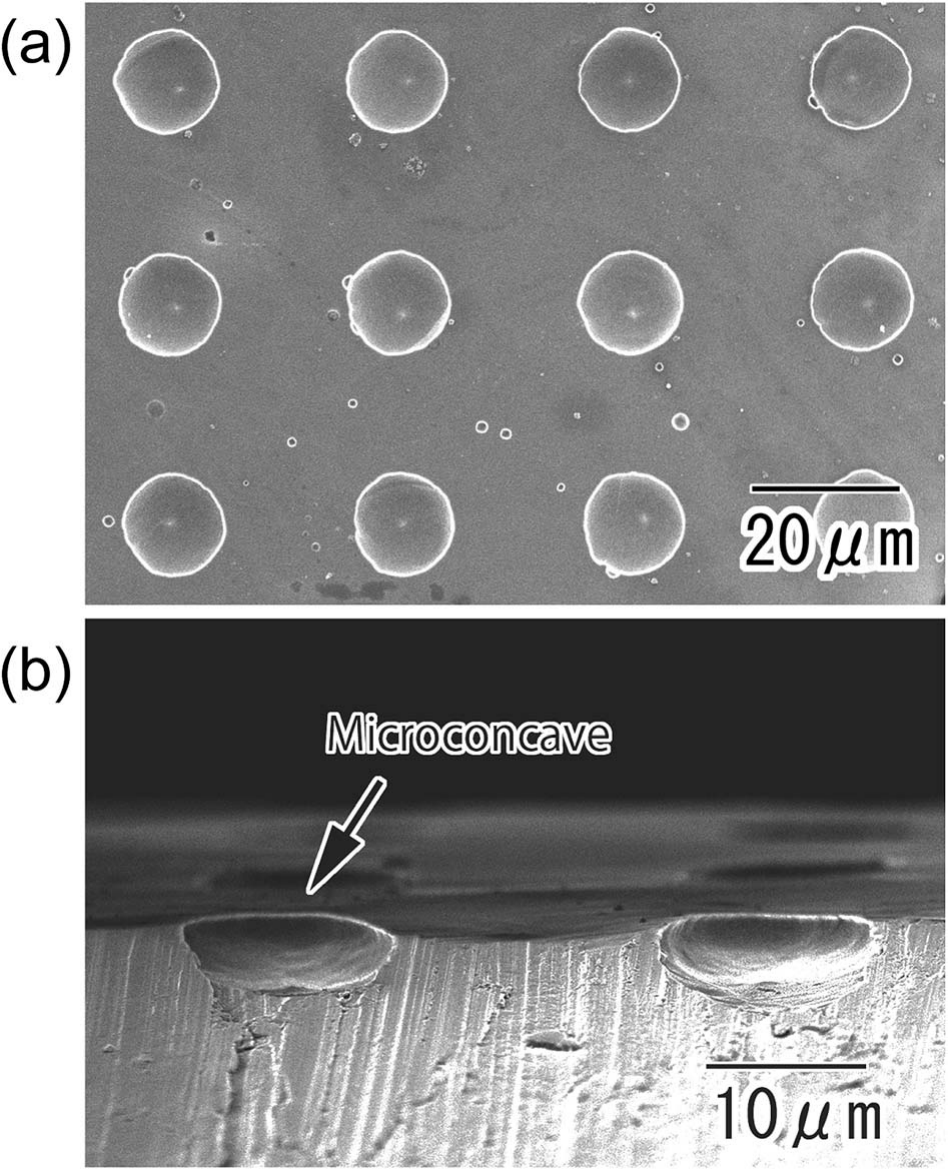
SEM images of micro-concave array formed on the surface of an Al substrate; (a) surface and (b) cross-sectional view.

Fig. 3 shows SEM images of an Al substrate with a micro-concave array obtained by removal of the oxide layer using a mixed solution of chromic acid and phosphoric acid. From the SEM image shown in Fig. 3(a), it was observed that micro-concaves of 16 μm diameter were ideally arranged with a 30 μm period. The cross-sectional image shown in Fig. 3(b) indicates that the micro-concaves were hemispherical and 4.5 μm in depth.

**Fig. 4:**
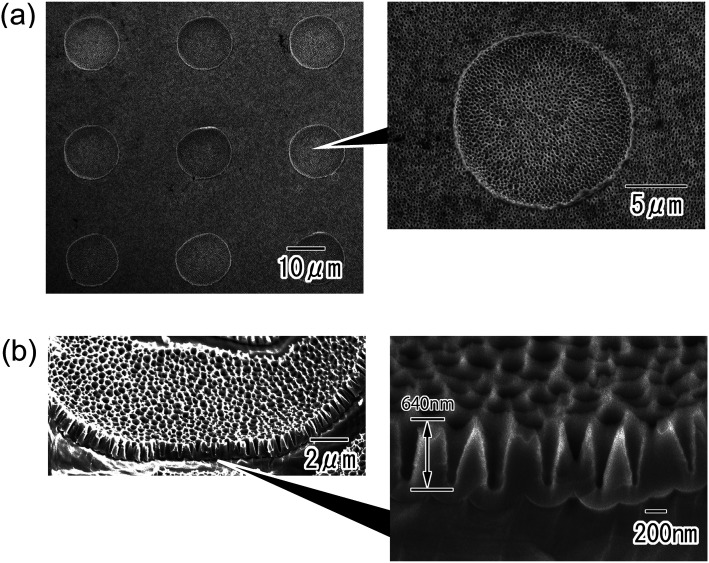
SEM images of anodic porous alumina molds with nano/micro hierarchical structures; (a) surface and (b) cross-sectional view.

Fig. 4 shows anodic porous alumina with the micro/nano hierarchical structure. The surface image in Fig. 4(a) shows that the micro-concave array was maintained even after anodization to form the nanohole array structure. A nanohole array structure with tapered holes was observed over the entire surface of the sample. The depth of the tapered nanoholes was found to be 640 nm from the cross-sectional SEM image shown in Fig. 4(b).

**Fig. 5:**
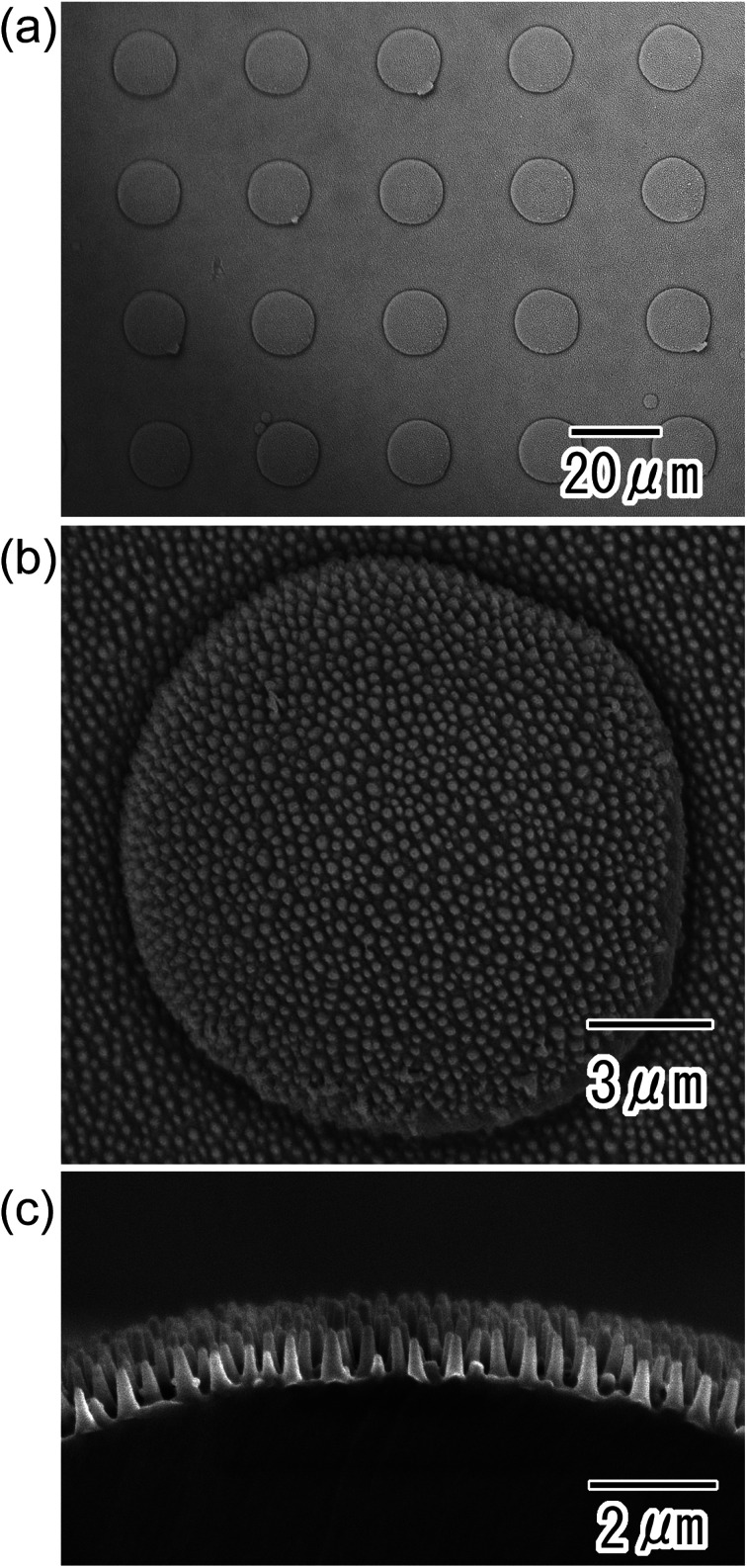
SEM images of polymer nano/micro hierarchical structures prepared by nanoimprinting using anodic porous alumina molds; (a) low-magnification view of surface, (b) high-magnification view of surface, and (c) cross-sectional view.

Fig. 5 shows the polymer micro/nano hierarchical structures prepared by nanoimprinting using the anodic porous alumina mold. The surface images shown in Fig. 5(a) and (b) confirmed that a hierarchical structure composed of nanopillars and micro-convexes was successfully formed on the surface of the PET sheet by the nanoimprinting. The cross-sectional image shown in Fig. 5(c) revealed that the nanopillars stood vertically on the curved surface of the micro-convexes.

**Fig. 6:**
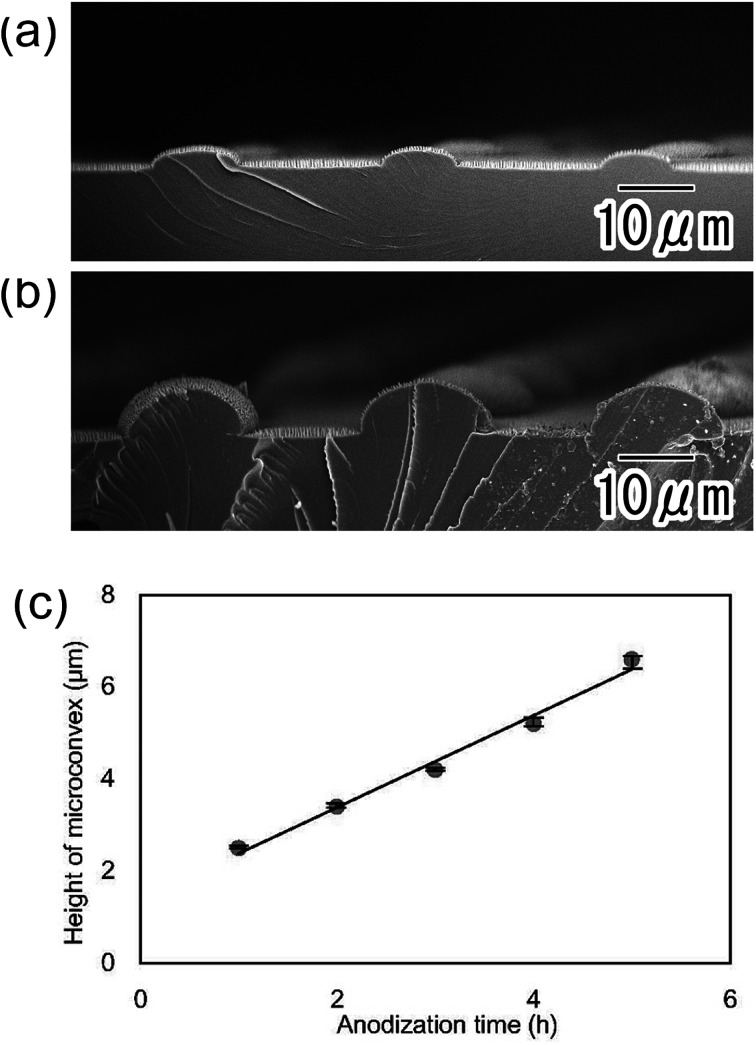
Cross-sectional SEM images of nano/microstructures with micro-convex heights of (a) 2.1 and (b) 6.5 μm. (c) Relationship between micro-convex height and anodization time to form hemispherical oxide layer.

In the present process, the height of the micro-convexes can be controlled by adjusting the anodization time in the preparation of the hemispherical oxide layer. Fig. 6(a) and (b) show SEM images of polymer hierarchical structures with heights of 2.1 and 6.5 μm, respectively. Fig. 6(c) shows the relationship between the micro-convex height and anodization time. The height of the polymer micro-convexes prepared by nanoimprinting showed a good linear relationship with the anodization time in the preparation of the hemispherical oxide layer.

**Fig. 7:**
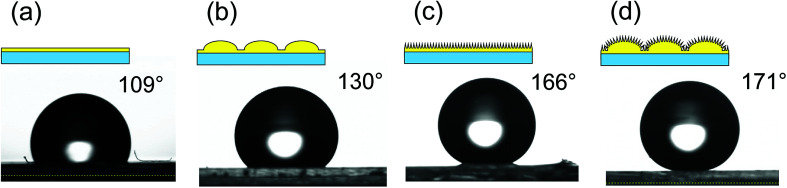
Schematic illustrations of the surfaces and photographs of water droplets on (a) smooth surface, (b) micro-convex array, (c) nanopillar array, and (d) nano/micro hierarchical structure.

The micro/nano hierarchical structures obtained by the present process can be applied to the fabrication of a superhydrophobic surface owing to their geometrical structure. Fig. 7 shows photographs of water droplets on (a) a smooth surface, (b) a micro-convex array, (c) a nanopillar array, and (d) a micro/nano hierarchically structured surface. Each surface was pretreated with a fluoroalkylsilane solution to form a hydrophobic coating, because the intrinsic contact angle of the photocurable monomer used for the nanoimprinting was 64°. The order of hydrophobicity was found to be hierarchical structure > nanopillar array > micro-convex array > smooth surface. Thus, the micro/nano hierarchical surface was the most effective as a water-repellent surface. There have been some reports that micro/nano hierarchical structures without hydrophobic treatment exhibit excellent hydrophobic properties.^28,29^ However, the hierarchical surface prepared by the present process did not exhibit superhydrophobic properties without hydrophobic treatment.

**Fig. 8:**
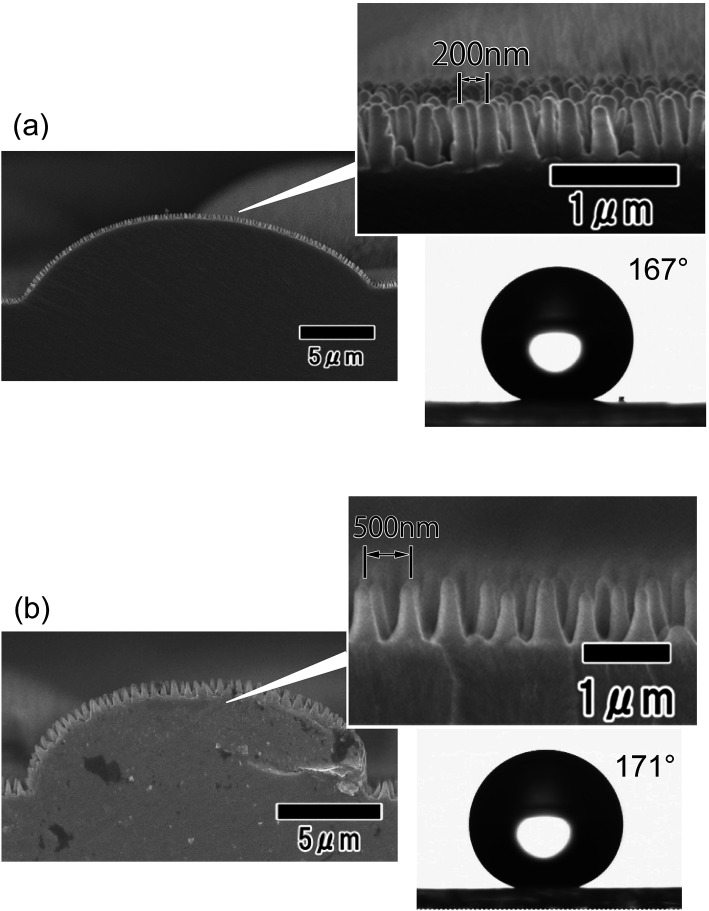
Cross-sectional SEM images of nano/micro hierarchical structures with nanopillar array periods of (a) 200 and (b) 500 nm and photographs of water droplets on their surfaces.

Fig. 8 shows the hydrophobic properties of hierarchical structures with different nanopillar periods. For this experiment, hierarchical structures with tapered nanopillar arrays having 200 and 500 nm periods were formed. The period of the nanopillars was controlled by adjusting the anodizing voltage of the mold. The static contact angles of water droplets were 167° and 170° for the arrays with 200 and 500 nm period, respectively. The hierarchical structure with nanopillars having a 500 nm period had slightly greater water repellency than the structure with a 200 nm period. For the case of a nanopillar array formed on a flat surface without micropatterns, it was confirmed that a nanopillar array with a larger period exhibits greater water repellency. Nanopillar array structures with larger periods have more trapped air between the nanopillars on their surface, and thus exhibit greater water repellency. This behavior can be explained by the Cassie–Baxter model, which describes the wetting of rough surfaces containing trapped air.^30,31^

**Fig. 9:**
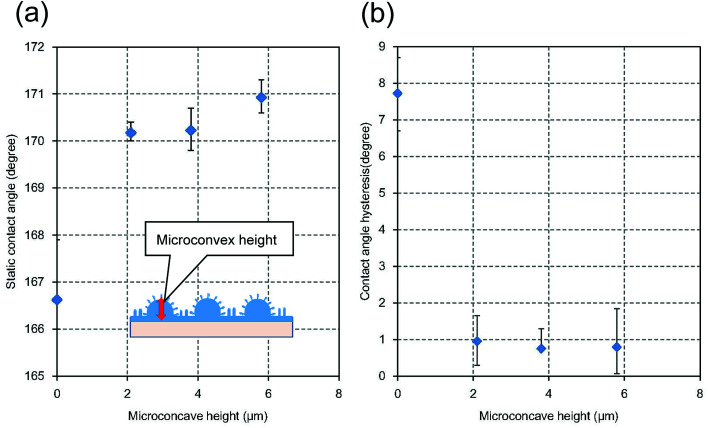
(a) Relationship between micro-convex height and static water droplet contact angle. (b) Relationship between micro-convex height and contact angle hysteresis.

The water repellency of the micro/nano hierarchical structures prepared by the present process also depended on their microstructure. Fig. 9(a) shows the static contact angles of hierarchical structures with different micro-convex height. The heights and period of the nanopillar array of all samples shown in Fig. 9 were 610 and 500 nm, respectively. The nano/micro hierarchical structures exhibited greater water repellency than the nanopillar array formed on the flat surface, which was indicated by the micro-convex height of 0 in Fig. 9(a). The static repellency of the hierarchical structure increased with increasing height of the micro-convexes. Fig. 9(b) shows the relationship between the micro-convex height and the contact angle hysteresis, which is defined as the difference between the advancing and receding contact angles. A low contact angle hysteresis implies a low adhesion force of water droplets on the surface of the substrate. The hierarchical surfaces exhibited lower contact angle hysteresis than the nanopillar array formed on the flat surface without a micropattern. The contact angle hysteresis of the hierarchical structures prepared by the present process was approximately 1°. The sliding angle of the hierarchical structures was also approximately 1°.

**Fig. 10:**
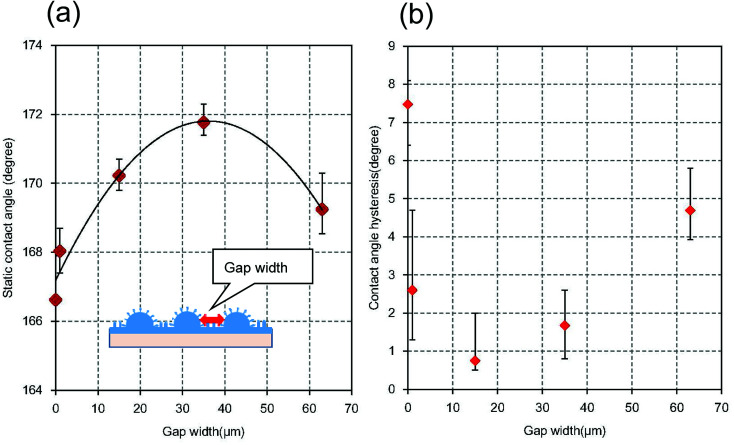
(a) Relationship between micro-convex gap width and static water droplet contact angle. The values of the gap width were 0, 1, 15, 35, and 63 μm, respectively. (b) Relationship between micro-convex gap width and contact angle hysteresis.

Fig. 10(a) shows the static contact angles of micro/nano hierarchical structures with different micro-convex gap widths. The width of the gap between the micro-convexes was controlled by changing the pattern of the resist mask. It was confirmed that the static contact angle increased with increasing micro-convex gap width up to 35 μm, then decreased at gap widths greater than 35 μm. This occurred because the water droplet sunk into the wide gap between the micro-convexes, which reduced the difference between the static contact angles of the hierarchical structure and nanopillar array structure. Fig. 10(b) shows the relationship between the micro-convex gap widths and the contact angle hysteresis. It was also observed that the contact angle hysteresis increased with increasing micro-convex gap width up to 35 μm.

## Conclusions

Polymer micro/nano hierarchical structures were prepared by nanoimprinting using anodic porous alumina molds. The shape of the nanopillars and micro-concaves in the hierarchical structure could be controlled by adjusting the preparation condition of the anodic porous alumina molds. Contact angle measurements revealed that the optimized hierarchical structures obtained by the present process exhibited superhydrophobicity with a contact angle of 171° and contact angle hysteresis of 1°. The present process is applicable to the high-throughput preparation of large superhydrophobic surfaces composed of micro/nano hierarchical structures.

## Conflicts of interest

There are no conflicts to declare.

## Supplementary Material
