# Correction: Preparation of superhydrophobic surfaces with micro/nano alumina molds

**DOI:** 10.1039/c8ra90084a

**Published:** 2018-11-12

**Authors:** Takashi Yanagishita, Kaito Murakoshi, Toshiaki Kondo, Hideki Masuda

**Affiliations:** Department of Applied Chemistry, Tokyo Metropolitan University 1-1 Minamiosawa Hachioji Tokyo 192-0397 Japan yanagish@tmu.ac.jp

## Abstract

Correction for ‘Preparation of superhydrophobic surfaces with micro/nano alumina molds’ by Takashi Yanagishita *et al.*, *RSC Adv.*, 2018, **8**, 36697–36704.

The authors regret that the title shown in the original article was incorrect. The correct title should read as follows:

Preparation of superhydrophobic surfaces with micro/nano hierarchical structures by nanoimprinting using anodic porous alumina molds.

The Royal Society of Chemistry apologises for these errors and any consequent inconvenience to authors and readers.

## Supplementary Material

